# The Effects of Perioperative Music Interventions in Pediatric Surgery: A Systematic Review and Meta-Analysis of Randomized Controlled Trials

**DOI:** 10.1371/journal.pone.0133608

**Published:** 2015-08-06

**Authors:** Marianne J. E. van der Heijden, Sadaf Oliai Araghi, Monique van Dijk, Johannes Jeekel, M. G. Myriam Hunink

**Affiliations:** 1 Department of Pediatrics, Erasmus MC, Rotterdam, the Netherlands; 2 Department of Neuroscience, Erasmus MC, Rotterdam, the Netherlands; 3 Department of Pediatrics, division of Neonatology and Department of Pediatric Surgery, Intensive care Erasmus MC, Rotterdam, the Netherlands; 4 Department of Epidemiology, Erasmus MC, Rotterdam, the Netherlands; 5 Department of Radiology, Erasmus MC, Rotterdam, the Netherlands; 6 Department of Health Policy and Management, Harvard T.H. Chan School of Public Health, Boston, Massachusetts, United States of America; Federal University of Rio de Janeiro, BRAZIL

## Abstract

**Objective:**

Music interventions are widely used, but have not yet gained a place in guidelines for pediatric surgery or pediatric anesthesia. In this systematic review and meta-analysis we examined the effects of music interventions on pain, anxiety and distress in children undergoing invasive surgery.

**Data Sources:**

We searched 25 electronic databases from their first available date until October 2014.

**Study Selection:**

Included were all randomized controlled trials with a parallel group, crossover or cluster design that included pediatric patients from 1 month to 18 years old undergoing minimally invasive or invasive surgical procedures, and receiving either live music therapy or recorded music.

**Data Extraction and Synthesis:**

4846 records were retrieved from the searches, 26 full text reports were evaluated and data was extracted by two independent investigators.

**Main Outcome Measures:**

Pain was measured with the Visual Analogue Scale, the Coloured Analogue Scale and the Facial Pain Scale. Anxiety and distress were measured with an emotional index scale (not validated), the Spielberger short State Trait Anxiety Inventory and a Facial Affective Scale.

**Results:**

Three RCTs were eligible for inclusion encompassing 196 orthopedic, cardiac and day surgery patients (age of 1 day to 18 years) receiving either live music therapy or recorded music. Overall a statistically significant positive effect was demonstrated on postoperative pain (SMD -1.07; 95%CI-2.08; -0.07) and on anxiety and distress (SMD -0.34 95% CI -0.66; -0.01 and SMD -0.50; 95% CI -0.84; - 0.16.

**Conclusions and Relevance:**

This systematic review and meta-analysis indicates that music interventions may have a statistically significant effect in reducing post-operative pain, anxiety and distress in children undergoing a surgical procedure. Evidence from this review and other reviews suggests music therapy may be considered for clinical use.

## Introduction

Adults and children undergoing surgery may experience perioperative pain, anxiety and distress[1]. Unfortunately it is not always possible to completely prevent postoperative pain with analgesics. Therefore there is an increasing interest in non-pharmacological interventions, among which music interventions. [2–4]

Roughly two types of music interventions are distinguished: live music therapy and recorded music. In live music therapy a trained music therapist plays music and applies various therapeutic techniques to reach a therapeutic goal. One of these techniques is known as music entrainment [5], in which the music therapist first uses music to match the patient’s physiological and emotional states and then gradually changes the music to modify the patient’s state. Recorded music on the other hand, implies listening to pre-recorded music selected by a music therapist, or by patients themselves provided they are old enough to do so[3].

Few studies have been performed on the effects of music interventions in children, and music interventions are not included in guidelines for pediatric surgery and anaesthesiology. However, music is used in clinical settings around the world [6] and is perceived to be a non-invasive, inexpensive and useful complementary intervention to reduce pain, anxiety and distress and to improve relaxation.

Our aim is to examine the effectiveness of music interventions to reduce pain, anxiety and distress in pediatric patients undergoing minimally invasive or invasive surgery through a systematic review and meta-analysis of the literature.

## Methods

This systematic review and meta-analysis was performed according to the recommendations of the Cochrane Collaboration as documented in our review protocol (see S1 File). For statistical analysis we used Review Manager (RevMan 5.2) (The Nordic Cochrane Centre, Copenhagen, Denmark, 2012). For assessing risk of bias we used the Cochrane Risk of Bias tool.

### Criteria for considering studies for this review

Inclusion criteria were all randomized controlled trials (RCT) with a parallel group, crossover or cluster design that included pediatric patients from 1 month to 18 years old undergoing minimally invasive or invasive surgical procedures. Studies were only included if patients received the music intervention before, during or after the surgical procedure and if outcomes were measured during or after the surgical procedure. Studies were only included if the control group received standard care, no music or another intervention. Music interventions could be live music therapy offered by a music therapist or recorded music.

Exclusion criteria were studies on multimodal interventions, in which music is offered in combination with other therapies such as massage. Excluded were studies on non-invasive surgery, neonates, adults, dental and ophthalmological surgical patients, non-randomized trials, papers not written in English, and narrative reviews. Auditory stimuli produced by non-human agents such as nature sounds or sounds like fixated beeps were excluded. Studies that performed the intervention pre-operatively and only measured outcomes prior to surgery were also excluded.

### Search methods for identification of studies

We searched 13 electronic databases and trial registers: 1. Cochrane Central Register of Controlled Trials (CENTRAL); 2. MEDLINE (Ovid) (1950 to present); 3. EMBASE (1980 to present); 4. CINAHL (1982 to present); 5. PsycINFO (1967 to present); 6. AMED (1985 to present); 7. Web of Science (1945 to present) 8.Scopus (1995 to present) 9. The specialist music therapy research database at www.musictherapyworld.net; 10. CAIRSS for Music; 11. ClinicalTrials.gov(http://www.clinicaltrials.gov/); 12. Current Controlled Trials (http://www.controlledtrials.com/); 13. National Research Register (http://www.updatesoftware.com/National/)

Furthermore we hand-searched 12 journals from their first available date until October 2014: 1. Australian Journal of Music Therapy; 2. Canadian Journal of Music Therapy; 3. The International Journal of the Arts in Medicine; 4. Journal of Music Therapy; 5. Journal for Art Therapies in Education, Welfare and Health Care; 6. Music Therapy; 7. Music Therapy Perspectives; 8. Nordic Journal of Music Therapy; 9. Music Therapy Today (online journal of music therapy); 10. Voices (online international journal of music therapy) 11. New Zealand Journal of Music Therapy; 12. British Journal of Music Therapy. We checked the reference lists of the most relevant articles (see S2 File for the full list of search terms and databases).

### Data collection

Two authors (MvdH and SO) selected the studies by scanning the titles and abstracts of all 4846 records retrieved from the searches. The study was rejected if the title or abstract clearly indicated that the trial did not meet the inclusion criteria. Out of the 4846 records, 26 full text reports were evaluated and data was extracted following the Cochrane guidelines by two independent investigators (MvdH and SO). Any disagreements between the two data extractors were resolved by discussions with two other authors (MvD and JJ). Two authors (MvdH and SO) emailed researchers (Nilsson) to make further inquiries about their study.

### Data analysis

All outcomes in this review are presented as continuous data. For all intervention and control groups we calculated intragroup mean differences (MD) with 95% confidence intervals (CI) comparing post versus pre intervention outcomes. Furthermore, intergroup differences were analyzed comparing the intervention and control group outcomes. Effect size was defined by Cohen’s rule-of-thumb: small effect is <0.2; moderate effect is 0.5 and large effect is >0.8.[7]

Comparable pain and distress outcome measures from the selected RCTs were used in a meta-analysis. For all outcome measures the intergroup standardized mean difference (SMD) with the corresponding 95% CI was calculated as effect size. Heterogeneity was determined by the I-squared (I^2^) statistic. Pooled estimates of the SMD were calculated using the random-effects model assuming that underlying heterogeneity exists, irrespective of whether the I^2^ statistic indicates heterogeneity, and to be conservative in our estimated 95% CI[8]. A forest plot analysis served to show the effects of music interventions on pain, anxiety and distress scores for the intervention and control groups.

Because the intervention used in one of the included studies consisted of a first and second live music intervention entrainment (one in the morning, one in the afternoon), these results were analyzed separately for the intergroup analysis [9]. However, in pooling the results, we could not use both entrainments because that would have duplicated the patients from this study. We decided to only use the results of the second music intervention entrainment because it was the most conservative estimate with the smallest reported effect.

## Results

An extensive search in 13 databases and 12 hand-searched journals resulted in 4846 records (See S3 File). Only 4 RCTs examining perioperative music interventions were identified. One was excluded because it did not match the inclusion criteria[10] (see S4 File for an overview of excluded articles). Table 1 gives an overview of the characteristics of the three included studies. These had a total of 196 participants, ranging in age from 1 day to 18 years old, were reported between 2006 and 2010 and carried out in the USA[9], Sweden[11] and Brazil[12]. Bradt et al included orthopaedic in-patients, Nilsson et al included patients undergoing minimally invasive day-surgery for miscellaneous conditions and Hatem et al included in-patients undergoing cardiac surgery [9, 11, 12].

**Table 1:** Characteristics of included studies.

Author, year, country	Patient population	Setting	N	Age mean (range)	Gender (%male)	Study design	Intervention (control)	Time and duration of music intervention	Outcome measurements	Time of measurement
Bradt (2010), USA	Orthopedic pediatric patients^1^	Two pediatric hospitals in Pennsylvania	32	14.2 (8–18 years)	56%	Cross-over RCT across 4 treatment sequences	Live music entrainment (no music)	Post-operative: 30–45 minutes	*Pain*: VAS (scale 0–10) self-report	*Pain*: Before, during, after intervention
									*Emotional state*: Bipolar descriptor (scale 0–5) self-report	*Emotional state*: Before, during, after intervention
Nilsson (2009), Sweden	Pediatric day surgery^2^	Queen Silvia Children’s Hospital, Gothenburg Academic hospital.	80	NR (7–16 years)	50%	Parallel group RCT 1:1	Recorded music MusiCure (no music)	Post-operative: Start at admission to PACU for 45 minutes	*Pain*: CAS (scale 0–10) self-report	*Pain*: Pre-operative and 1h after PACU
									*Distress*: FAS (scale 0–10) self-report	*Distress*: Pre-operative, in the PACU and 1h after PACU
									*Anxiety*: STAI (scale 6–24) self-report	*Anxiety*: Pre-operative, in the PACU and 1h after PACU
									*Morphine administration*: FLACC (scale 0–10) by nurse	*Morphine administration*: Every 15 minutes during stay in PACU and before the child left the PACU
Hatem (2006), Brazil	ICU pediatric carciac patients^3^	Hospital do Coracao	84	NR (1 day– 16 years)	NR	Parallel group RCT 3.4: 1	Recorded music Vivaldi’s Four Seasons (no music)	Post-operative: 30 minutes after surgery for 30 minutes	*Pain*: FAS (scale 0–10) by nurse	*Pain*: First and last minutes of the intervention
									*Vital signs*: BP, DBP, HR, IQ, MBP, RR, SBP, SatO2, T by nurse	*Vital signs*: Before intervention and 30 minutes after intervention

^1^.spine fusion, centralization of wrist, scar revision, tibial rodding, osteotomy and placement of external fixator, osteotomy and leg lengthening, pectus repair, hardware removal.

^2^.Arthroscopy, endoscopy, extraction of pain/nail/thread, hernia/hydrocele, superficial surgery.

^3^.acyanotic congenital heart disease (ACHD) with left-right shunt; obstructive ACHD, cyanotic congenital heart disease (CCHD) with pulmonary hypoflow; CCHD with pulmonary hyperflow, complex congenital heart disease (CHD) and acquired heart diseases.

In all three studies the music interventions were performed post-operatively and all evaluated the effects of music on the patient after surgery comparing the outcome to the baseline measurement and to the control group. Medical conditions or the complexity of the surgery were not considered as possible confounding variables due to the paucity of data which precluded meaningful analysis of these variables. One study evaluated the effects of live music therapy (music entrainment) in a cross-over design[9]; two studies performed a parallel group RCT on the effects of a recorded music intervention (MusiCure and Vivaldi’s Four Seasons, respectively) [11, 12] (see Table 1).

### Risk of bias

We have used the Cochrane Handbook for Systematic reviews of Interventions to assess the risk of bias of the included studies. The overall risk of bias was moderate (see S5 File). Nilsson used an appropriate method of allocation by using opaque envelopes[11], Bradt et al used the drawing of lots, and Hatem et al assigned three consecutive participants to the intervention group and one to the control group[9, 12]. Only Nilsson et al and Hatem et al reported their power and sample size calculations[11, 12]. It was not clear if researchers were blinded for group allocation.

### Outcome measurements

#### Primary outcome: pain intensity

Across the studies pain intensity was measured with the Visual Analogue Scale (VAS), Coloured Analogue Scale (CAS) and the Facial Pain Scale (FPS) [9, 11, 12]. In Bradt et al the patients self-reported pain intensity with the VAS[9] before, during and after the music intervention. Nilsson et al assessed self-reported pain intensity by CAS preoperatively, at the arrival to the Post Anaesthesia Care Unit (PACU) and one hour after the PACU[11]. In the study of Hatem et al the Facial Pain Scale was assessed by a nurse during the first and last minutes of the music intervention[12] (see Table 1).

#### Secondary outcome: anxiety and distress descriptors

As a secondary outcome, two out of the three studies measured anxiety and distress descriptors[9, 11]. Bradt et al used a 5-point scale with 8 bipolar descriptor items to measure the participants’ emotional state. Each of the items was given a numerical value from 1 ‘very negative’ to 5 ‘very positive’. This emotional index scale was developed by Bradt et al and was not validated.

To measure anxiety Nilsson et al used the Spiegelberger short- State Trait Anxiety Inventory (STAI) on a scale of 6–24 points, which was not validated in children. The children filled in the short form of STAI preoperatively and 1 hour after the PACU. A Facial Affective Scale (FAS) was used to measure distress at the same time points as pain.

### Outcomes

**Fig 1:**
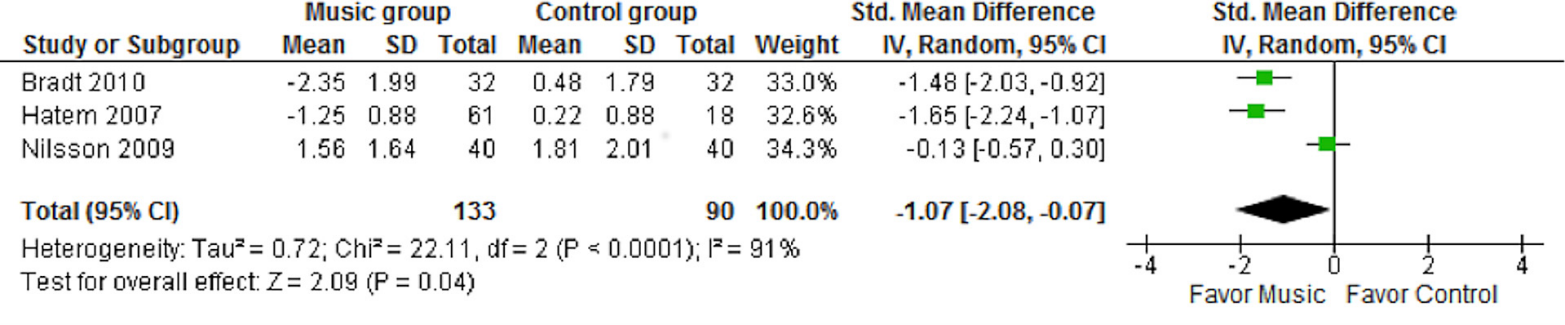
Pain change score (music vs. no music) before and after the intervention by CAS and FAS

**Fig 2:**
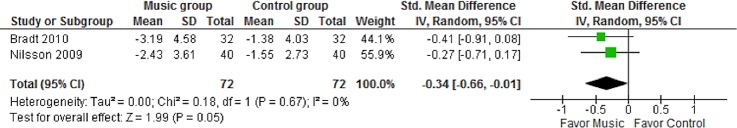
Anxiety/distress change score (music vs. no music) before and after the intervention measured by Short-STAI and bipolar descriptors.

**Fig 3:**
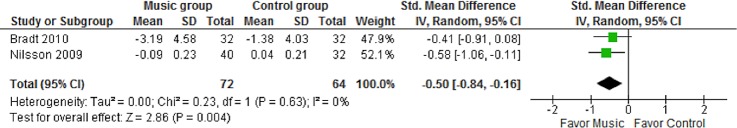
Anxiety/distress change score (music vs. no music) before and after the intervention measured by FAS and bipolar descriptors.

Table 2 provides the intragroup results of all the primary and secondary outcomes reported in the included studies. All three studies show statistically significant intragroup improvements for pain and anxiety and distress descriptors (Table 2). Table 3 and Figs 1–3 provide the comparison between the intervention and control groups for pain and anxiety and distress descriptors.

**Table 2:** Intragroup comparisons of post music intervention versus baseline.

Scale (outcome)		N	MD	SD	95% CI	P value
Bradt (2010)	VAS_E1_ (pain)	32	- 2.97	2.09	[-3.72; -2.22]	<0.001
	VAS_E2_ (pain)	32	- 2.35	1.99	[-3.07; -1.63]	<0.001
	VAS_C_ (pain)	32	0.48	1.79	[-0.17; 1.13]	0.14
	Emotional State__E1_ (anxiety)	32	- 6.16	6.67	[-8.56; -3.76]	<0.001
	Emotional State__E2_ (anxiety)	32	- 3.19	4.58	[-4.84; -1.54]	<0.001
	Emotional State__CMorning_(anxiety)	32	3.69	2.97	[2.63; 4.75]	<0.001
	Emotional State__CAfternoon_(anxiety)	32	- 1.38	4.03	[-2.83; 0.07]	0.06
Nilsson (2009)	CAS_Intervention_(pain)	40	1.56	1.64	[1.04; 2.08]	<0.001
	CAS_Control_(pain)	40	1.81	2.01	[1.17; 2.45]	<0.001
	STAI _Intervention_ (anxiety)	40	- 2.43	3.61	[-3.58; -1.28]	<0.001
	STAI _Control_ (anxiety)	40	- 1.55	2.73	[-2.42; -0.68]	<0.001
	FAS _Intervention_ (anxiety)	40	- 0.09	0.23	[-0.16; -0.02]	0.02
	FAS _Control_ (anxiety)	40	0.04	0.21	[-0.03; 0.11]	0.24
Hatem (2006)	FAS _Intervention_ (pain)	61	- 1.25	0.88	[-1.48; -1.02]	<0.001
	FAS _Control_ (pain)	18	0.22	0.88	[-0.22; 0.66]	0.30

VAS: Visual Analogue Scale.

CAS: Coloured Analogue Scale.

STAI: Spielberger short-State Trait Anxiety Inventory.

FAS: Facial Affective Scale.

MD: mean difference.

E1: first entrainment.

E2: second entrainment.

C: Control group.

Negative MD: decreased mean difference.

Positive MD: increased mean difference.

95% CI of the MD: Confidence Interval.

SD: Standard deviation.

**Table 3:** Intergroup comparisons of music intervention versus control.

	Scale (outcome)	N	MD	95% CI *	SE	SMD	95% CI **	P value
**Bradt (2010)**	VAS_E2_ (pain)	32	-2.83	[-3.76to -1.90]	0.47	-1.48	[-2.03; -0.92]	<0.001
	Emotional State__E2Afternoon_ (anxiety)	32	-1.81	[-3.92to 0.30]	1.08	-0.41	[-0.91; 0.08]	0.10
**Nilsson (2009)**	CAS (pain)	80	-0.25	[-1.054; 2.45]	0.41	-0.13	[-0.57; 0.30]	0.54
	STAI (anxiety)	80	-0.88	[-2.28; 0.52]	0.72	-0.27	[-0.71; 0.17]	0.22
	FAS (anxiety)	80	-0.13	[-0.23; -0.03]	0.05	-0.58	[-1.06; -0.11]	0.02
**Hatem (2006)**	FAS (pain)	79	-1.47	[-1.93; -1.01]	0.24	-1.65	[-2.24; -1.07]	<0.001

MD: mean difference.

SMD: Standardized mean difference.

*95% CI of the MD.

**95% CI of the SMD.

E1: first entrainment.

E2: second entrainment.

C: Control group.

Negative (S)MD: decreased (Standardized) Mean Difference.

Positive (S)MD: increased (Standardized) Mean Difference.

Pain scores (Fig 1) demonstrated significant heterogeneity (Chi^2^ 22.11, I^2^ = 91%, (P<0.0001)) across studies. The random effects pooled result showed a statistically significant standardized mean difference of -1.07 [95% CI -2.08 to -0.07] between the intervention and control group in favour of music.

Anxiety scores (Fig 2) by Short-STAI and bipolar descriptors demonstrated no statistically significant heterogeneity (Chi^2^ 0.18, I^2^ = 0%, (P = 0.67)). The standardized mean difference of anxiety and distress between the intervention and control group was -0.34 [95% CI -0.66 to -0.01] in favour of music.

Anxiety and distress scores (Fig 3) by FAS and bipolar descriptors demonstrated no statistically significant heterogeneity (Chi^2^ 0.23, I^2^ = 0%, (P = 0.63)). The standardized mean difference of anxiety between the intervention and control group was -0.50 [95% CI -0.84 to -0.16] in favour of music.

## Discussion

The aim of this systematic review was to investigate the effect of perioperative music interventions in children undergoing surgical procedures.

Two studies reported a large significant pain-reducing effect and one study a small non-significant pain-reducing effect of music between the intervention and control group. Comparing before and after the intervention within the intervention groups, all studies showed a large and significant decline in pain, anxiety and distress descriptors.

The present review is the first on this topic that strictly adheres to the methods recommended in the Cochrane Guidelines for writing a Systematic Review[8]. The findings should be interpreted in the light of its limitations, most of which are related to the original studies. First, the overall risk of bias was moderate. Second, there was heterogeneity in the types of music interventions, the type of surgery across studies, patient populations and outcome measures.

Although the heterogeneity between the studies is a limitation, we were able to calculate the standardized mean difference per group and to pool the results for the pain and anxiety and distress descriptor outcomes. Ideally, we would have tried to adjust for the heterogeneity by performing a meta-regression analysis or subgroup analysis, but the number of studies was insufficient to perform such analyses. The variability in treatment effect across studies is likely to be due to the above-mentioned heterogeneity in the types of music interventions, the type of surgery across studies, patient populations and outcome measures.

Although only three studies could be included in this meta-analysis, the results show a significant reduction of pain, anxiety and distress descriptors in pediatric surgical patients. Similar results have been found in other patient populations. Thirteen Cochrane systematic reviews have been published on music interventions in adults for various indications [3, 6, 13–23]. All reported positive effects of music on anxiety and distress, pain and quality of life, although it was noted that the general methodological quality of reviewed studies was moderate to low. Furthermore authors recommended exploring possibly differential effects of live music therapy versus recorded music interventions. Apart from the Cochrane reviews, thirty descriptive and systematic reviews on the effects of music interventions on perioperative pain and anxiety in adults were published[2, 24–40]. Together the body of evidence suggests that music therapy in the perioperative setting has the potential to positively affect pain outcomes, anxiety and distress.

For future research we would like to stress the importance of rigorous study protocols, the use of larger sample sizes and validated outcome measures. For research in children, we would recommend to pay heed to the Consensus Statement of McGrath et al. regarding appropriate outcomes measurements in pain research.[41]

Study populations should be more homogenous in terms of age and type of procedure. Observer bias could perhaps be prevented by recording the patient on video while receiving the intervention, blind the video images for the allocated intervention and have independent assessors score the outcome measures using validated measurements while watching the recordings[4].

Furthermore, we would like to suggest cost-effectiveness studies comparing live music therapy with recorded music. Apart from the possibly different effects of live music therapy versus recorded music, the timing of the intervention and the effect of self-selected versus therapist selected music deserve attention[3].

This review shows that few RCTs have been performed on effects of music in pediatric patients undergoing surgery, but that music interventions are worthwhile to further investigate for its clinical usefulness. State-of-the-art RCTs evaluating music interventions are difficult to perform in particular due to the inherent performance bias and detection bias. The only way to perform a double-blinded study is to offer recorded music through headphones to patients under general anesthesia which would preclude evaluation of the potential beneficial effect of music pre- and post surgery[42]. Furthermore it is impossible to blind patients for live music therapy by a music therapist.

In conclusion, this review shows that music as a non-pharmacological adjuvant intervention has potential in reducing pain, anxiety and distress in children undergoing surgery. Its non-invasive nature is an advantage.

## Supporting Information

S1 File Review protocol. (DOC)

S2 File Full list of search terms and databases. (DOC)

S3 File Prisma Checklist and flowchart. (DOC)

S4 File Excluded articles. (DOC)

S5 File Risk of bias. Quality assessment of studies.
(DOC)
