# Supplementary material for: The Effects of Perioperative Music Interventions in Pediatric Surgery: A Systematic Review and Meta-Analysis of Randomized Controlled Trials
Source: PLoS One. 2015 Aug 6;10(8):e0133608. doi: 10.1371/journal.pone.0133608 (PMC4527726; doi:10.1371/journal.pone.0133608)
Supplement: S1 File — (DOC) [file pone.0133608.s001.doc]

**S1 review protocol**

**Title:** The effects of perioperative music interventions in paediatric surgery: a systematic review and meta-analysis of randomized controlled trials. Review Protocol

**Objective**: What is known about the effectiveness of music interventions to reduce pain, anxiety and distress in paediatric patients undergoing minimally invasive or invasive surgery.

**Selection criteria: patients**

*Inclusion:* All pediatric patients (<18 years old), inpatients and outpatients, emergency and non-emergency undergoing invasive and non-invasive surgical procedures.

*Exclusion:* Neonates (till 28 days), adults, ventilated, dental and ophthalmological surgical patients

**Selection criteria: Intervention**

*Inclusion:* Music interventions delivered in a hospital setting. Live music therapy offered by a music therapist or recorded music, instrumental music, music with song. All type of interventionists. Individual delivery and group delivery (measured individually).

*Exclusion:* Multimodal interventions in which music is offered in combination with another therapy (example: massage). Auditory stimuli produced by non-human agents such as nature sounds or sounds like fixated beeps.

**Selection criteria: Comparison**

*Inclusion:* Standard care, no music or other interventions.

**Selection criteria: Outcome**

*Inclusion:* Interventions offered before during or after the surgical procedure, but only if the outcomes were measured during or after the surgical procedure. Pain, anxiety and distress outcomes

*Exclusion:* Studies that offered the intervention pre-operatively but only measured the outcomes prior to surgery.

**Selection criteria: Time frame**

No restriction on date of publication, all sources searched from their first available date.

**Selection criteria: Study design**

*Inclusion:* All randomized controlled trials with a parallel group, cross-over or cluster design

*Exclusion:* Non-randomized trials, papers not written in English, narrative reviews

**Search strategy**

All medical journals and music therapy journals (electronic and print). All databases available.

**Data extraction**

Data will be extracted by two researchers: Marianne van der Heijden (MvdH) and Sadaf Oliai Araghi (S.O.). Any disagreements regarding the data extraction will be resolved by Monique van Dijk (MvD), Hans Jeekel (JJ) and Myriam Hunink (MH). The Cochrane guidelines for Systematic Review will be followed.
