# Supplementary material for: The Effects of Perioperative Music Interventions in Pediatric Surgery: A Systematic Review and Meta-Analysis of Randomized Controlled Trials
Source: PLoS One. 2015 Aug 6;10(8):e0133608. doi: 10.1371/journal.pone.0133608 (PMC4527726; doi:10.1371/journal.pone.0133608)
Supplement: S2 File — (DOC) [file pone.0133608.s002.doc]

**S2 Full list of search terms and databases**

**Search terms**

**Embase**

(music/de OR 'music therapy'/de OR (music OR musical OR musicotherap*):ab,ti) AND (surgery/exp OR 'obstetric operation'/exp OR 'postoperative complication'/exp OR 'anesthesiological procedure'/exp OR 'perioperative nursing'/de OR 'postanesthesia nursing'/de OR 'operating room'/de OR 'recovery room'/de OR 'operating room personnel'/de OR (surger* OR surgic* OR peroperat* OR perioperat* OR preoperat* OR postoperat* OR operati* OR interoperat* OR intraoperat* OR anesthe* OR anaesthe* OR perianesthe* OR peranesthe* OR perianaesthe* OR peranaesthe* OR preanasthe* OR preanaesthe* OR postanasthe* OR postanaesthe*):ab,ti OR surgery:lnk)

**Medline OvidSP**

(music/ OR "music therapy"/ OR (music OR musical OR musicotherap*).ab,ti.) AND (exp "Surgical Procedures, Operative"/ OR exp "postoperative complications"/ OR "Anesthesiology"/ OR "perioperative nursing"/ OR "Operating Rooms"/ OR "recovery room"/ OR (surger* OR surgic* OR peroperat* OR perioperat* OR preoperat* OR postoperat* OR operati* OR interoperat* OR intraoperat* OR anesthe* OR anaesthe* OR perianesthe* OR peranesthe* OR perianaesthe* OR peranaesthe* OR preanasthe* OR preanaesthe* OR postanasthe* OR postanaesthe*).ab,ti. OR surgery.xs.)

**Cochrane central**

((music OR musical OR musicotherap*):ab,ti) AND ((surger* OR surgic* OR peroperat* OR perioperat* OR preoperat* OR postoperat* OR operati* OR interoperat* OR intraoperat* OR anesthe* OR anaesthe* OR perianesthe* OR peranesthe* OR perianaesthe* OR peranaesthe* OR preanasthe* OR preanaesthe* OR postanasthe* OR postanaesthe*):ab,ti)

**Web-of-science**

TS=(((music OR musical OR musicotherap*)) AND ((surger* OR surgic* OR peroperat* OR perioperat* OR preoperat* OR postoperat* OR operati* OR interoperat* OR intraoperat* OR anesthe* OR anaesthe* OR perianesthe* OR peranesthe* OR perianaesthe* OR peranaesthe* OR preanasthe* OR preanaesthe* OR postanasthe* OR postanaesthe*)))

**Scopus**

TITLE-ABS-KEY((music OR musical OR musicotherap*) AND (surger* OR surgic* OR peroperat* OR perioperat* OR preoperat* OR postoperat* OR operati* OR interoperat* OR intraoperat* OR anesthe* OR anaesthe* OR perianesthe* OR peranesthe* OR perianaesthe* OR peranaesthe* OR preanasthe* OR preanaesthe* OR postanasthe* OR postanaesthe*))

**PsycINFO OvidSP**

(music/ OR "music therapy"/ OR (music OR musical OR musicotherap*).ab,ti.) AND (exp "Surgery"/ OR "Surgical Patients"/ OR exp "Postsurgical Complications"/OR exp "Surgical Complications"/ OR "Anesthesiology"/ OR (surger* OR surgic* OR peroperat* OR perioperat* OR preoperat* OR postoperat* OR operati* OR interoperat* OR intraoperat* OR anesthe* OR anaesthe* OR perianesthe* OR peranesthe* OR perianaesthe* OR peranaesthe* OR preanasthe* OR preanaesthe* OR postanasthe* OR postanaesthe*).ab,ti.)

**PubMed publisher**

(music[tiab] OR musical[tiab] OR musicotherap*[tiab]) AND (surger*[tiab] OR surgic*[tiab] OR peroperat*[tiab] OR perioperat*[tiab] OR preoperat*[tiab] OR postoperat*[tiab] OR operati*[tiab] OR interoperat*[tiab] OR intraoperat*[tiab] OR anesthe*[tiab] OR anaesthe*[tiab] OR perianesthe*[tiab] OR peranesthe*[tiab] OR perianaesthe*[tiab] OR peranaesthe*[tiab] OR preanasthe*[tiab] OR preanaesthe*[tiab] OR postanasthe*[tiab] OR postanaesthe*[tiab]) AND publisher[sb]

**Cinahl**

(MH music+ OR MH "music therapy"+ OR TX (music OR musical OR musicotherap*)) AND (MH "Surgery, Operative"+ OR MH "postoperative complications"+ OR MH "Anesthesiology"+ OR MH "perioperative nursing"+ OR MH "Operating Rooms"+ OR MH "Post Anesthesia Care Units"+ OR TX (surger* OR surgic* OR peroperat* OR perioperat* OR preoperat* OR postoperat* OR operati* OR interoperat* OR intraoperat* OR anesthe* OR anaesthe* OR perianesthe* OR peranesthe* OR perianaesthe* OR peranaesthe* OR preanasthe* OR preanaesthe* OR postanasthe* OR postanaesthe*))

**Amed OvidSP**

(music OR musical OR musicotherap*) AND (surger* OR surgic* OR peroperat* OR perioperat* OR preoperat* OR postoperat* OR operati* OR interoperat* OR intraoperat* OR anesthe* OR anaesthe* OR perianesthe* OR peranesthe* OR perianaesthe* OR peranaesthe* OR preanasthe* OR preanaesthe* OR postanasthe* OR postanaesthe*)

**Handsearch**

(surger* OR surgic* OR peroperat* OR perioperat* OR preoperat* OR postoperat* OR operati* OR interoperat* OR intraoperat* OR anesthe* OR anaesthe* OR perianesthe* OR peranesthe* OR perianaesthe* OR peranaesthe* OR preanasthe* OR preanaesthe* OR postanasthe* OR postanaesthe*)

**Databases**: 1. Cochrane Central Register of Controlled Trials (CENTRAL); 2. MEDLINE (Ovid) (1950 to present); 3. EMBASE (1980 to present); 4. CINAHL (1982 to present); 5. PsycINFO (1967 to present); 6. AMED (1985 to present); 7. Web of Science (1945 to present) 8.Scopus (1995 to present) 9. The specialist music therapy research database at [www.musictherapyworld.net](http://www.musictherapyworld.net); 10. CAIRSS for Music; 11. ClinicalTrials.gov(<http://www.clinicaltrials.gov/>); 12. Current Controlled Trials (<http://www.controlledtrials.com/> ); 13. National Research Register ([http://www.updatesoftware.com/Nationa l/](http://livepage.apple.com/) )

Furthermore we hand-searched 12 journals from their first available date: 1. Australian Journal of Music Therapy; 2. Canadian Journal of Music Therapy; 3. The International Journal of the Arts in Medicine; 4. Journal of Music Therapy; 5. Journal for Art Therapies in Education, Welfare and Health Care; 6. Music Therapy; 7. Music Therapy Perspectives; 8. Nordic Journal of Music Therapy; 9. Music Therapy Today (online journal of music therapy); 10. Voices (online international journal of music therapy) 11. New Zealand Journal of Music Therapy; 12. British Journal of Music Therapy. See appendix 1 for the search terms. A recent search update was performed in October 2014.
