# Supplementary material for: The Effects of Perioperative Music Interventions in Pediatric Surgery: A Systematic Review and Meta-Analysis of Randomized Controlled Trials
Source: PLoS One. 2015 Aug 6;10(8):e0133608. doi: 10.1371/journal.pone.0133608 (PMC4527726; doi:10.1371/journal.pone.0133608)
Supplement: S3 File — (DOC) [file pone.0133608.s003.doc]

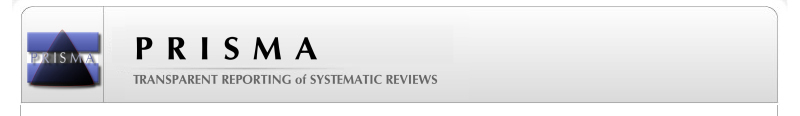
**PRISMA 2009 Flow Diagram**

**Screening**

**Included**

**Eligibility**

**Identification**

Records identified through database searching
(n = 4846)

Additional records identified through other sources
(n = 0)

Records excluded
(n = 4820)

4601 Non-RCT

130 RCTs in adults

85 Reviews

4 non-English

Full-text articles assessed for eligibility
(n = 26)

Full-text articles excluded, with reasons
(n = 23)

17 Non-invasive surgery

5 Study population not meeting inclusion criteria

1 Pre-operative outcome measurement only

Studies included in qualitative synthesis
(n = 3)

Studies included in quantitative synthesis (meta-analysis)
(n = 3)
