# Supplementary material for: The Effects of Perioperative Music Interventions in Pediatric Surgery: A Systematic Review and Meta-Analysis of Randomized Controlled Trials
Source: PLoS One. 2015 Aug 6;10(8):e0133608. doi: 10.1371/journal.pone.0133608 (PMC4527726; doi:10.1371/journal.pone.0133608)
Supplement: S4 File — (DOC) [file pone.0133608.s004.doc]

One study was excluded because the interventions and measurements only took place before surgery.^1^

Seventeen studies were excluded because they included non-invasive (surgical) procedures.^2-18^

Five studies were excluded because the study population did not meet the inclusion criteria.^19-23^

**1.** Kain ZN, Caldwell-Andrews AA, Krivutza DM, et al. Interactive music therapy as a treatment for preoperative anxiety in children: a randomized controlled trial. *Anesth Analg.* May 2004;98(5):1260-1266, table of contents.

**2.** Nguyen TN, Nilsson S, Hellstrom AL, Bengtson A. Music therapy to reduce pain and anxiety in children with cancer undergoing lumbar puncture: a randomized clinical trial. *J Pediatr Oncol Nurs.* May-Jun 2010;27(3):146-155.

**3.** Balan R, Bavdekar SB, Jadhav S. Can Indian classical instrumental music reduce pain felt during venepuncture? *Indian J Pediatr.* May 2009;76(5):469-473.

**4.** Windich-Biermeier A, Sjoberg I, Dale JC, Eshelman D, Guzzetta CE. Effects of distraction on pain, fear, and distress during venous port access and venipuncture in children and adolescents with cancer. *J Pediatr Oncol Nurs.* Jan-Feb 2007;24(1):8-19.

**5.** Caprilli S, Anastasi F, Grotto RP, Scollo Abeti M, Messeri A. Interactive music as a treatment for pain and stress in children during venipuncture: a randomized prospective study. *J Dev Behav Pediatr.* Oct 2007;28(5):399-403.

**6.** Liu RW, Mehta P, Fortuna S, et al. A randomized prospective study of music therapy for reducing anxiety during cast room procedures. *J Pediatr Orthop.* Oct-Nov 2007;27(7):831-833.

**7.** Kain ZN, Wang SM, Mayes LC, Krivutza DM, Teague BA. Sensory stimuli and anxiety in children undergoing surgery: a randomized, controlled trial. *Anesth Analg.* Apr 2001;92(4):897-903.

**8.** Fratianne RB, Prensner JD, Huston MJ, Super DM, Yowler CJ, Standley JM. The effect of music-based imagery and musical alternate engagement on the burn debridement process. *J Burn Care Rehabil.* Jan-Feb 2001;22(1):47-53.

**9.** Loewy J, Hallan C, Friedman E, Martinez C. Sleep/sedation in children undergoing EEG testing: a comparison of chloral hydrate and music therapy. *J Perianesth Nurs.* Oct 2005;20(5):323-332.

**10.** Noguchi LK. The effect of music versus nonmusic on behavioral signs of distress and self-report of pain in pediatric injection patients. *J Music Ther.* Spring 2006;43(1):16-38.

**11.** Kim SJ, Oh YJ, Kim KJ, Kwak YL, Na S. The effect of recorded maternal voice on perioperative anxiety and emergence in children. *Anaesth Intensive Care.* Nov 2010;38(6):1064-1069.

**12.** Padmanabhan R, Hildreth AJ, Laws D. A prospective, randomised, controlled study examining binaural beat audio and pre-operative anxiety in patients undergoing general anaesthesia for day case surgery. *Anaesthesia.* Sep 2005;60(9):874-877.

**13.** Huth MM, Broome ME, Good M. Imagery reduces children's post-operative pain. *Pain.* Jul 2004;110(1-2):439-448.

**14.** Hartling L, Newton AS, Liang Y, et al. Music to reduce pain and distress in the pediatric emergency department: a randomized clinical trial. *JAMA Pediatr.* Sep 2013;167(9):826-835.

**15.** Fanurik D, Koh JL, Schmitz ML. Distraction techniques combined with EMLA: Effects on IV insertion pain and distress in children. *Children's Health Care.* 2000;29(2):87 - 101.

**16.** Press J, Y. G, Maimon M, Gonen A, Goldman V, Buskila D. Effects of active distraction on pain of children undergoing venipuncture. *The Pain Clinic.* 2003;15(3):261.

**17.** Bufalini A. [Role of interactive music in oncological pediatric patients undergoing painful procedures]. *Minerva Pediatr.* Aug 2009;61(4):379-389.

**18.** Shahabi M, Kalani-Tehrani D, Eghbal M, Alavi-Majd H, Abed Saidi J. Comparing the effects of EMLA ointment with a diversionary activity (music) on vein puncture pain in school-age children. *Journal of Shahid Beheshti School of Nursing and Midwifery.* 2007;17(56).

**19.** Bansal P, Kharod U, Patel P, Sanwatsarkar S, Patel H, Kamar H. The effect of music therapy on sedative requirements and haemodynamic parameters under spiral anaesthesia: a prospective study. *Journal of Clinical and Diagnostic Research.* 2010;4(August):2782 - 2789.

**20.** Whitehead-Pleaux AM, Baryza MJ, Sheridan RL. The effects of music therapy on pediatric patients' pain and anxiety during donor site dressing change. *J Music Ther.* Summer 2006;43(2):136-153.

**21.** Burns DS, Robb SL, Haase JE. Exploring the feasibility of a therapeutic music video intervention in adolescents and young adults during stem-cell transplantation. *Cancer Nurs.* Sep-Oct 2009;32(5):E8-E16.

**22.** Lee DW, Chan AC, Wong SK, et al. Can visual distraction decrease the dose of patient-controlled sedation required during colonoscopy? A prospective randomized controlled trial. *Endoscopy.* Mar 2004;36(3):197-201.

**23.** Bradt J. The effects of music entrainment on postoperative pain perception in pediatric patients. *Music and Medicine.* 2010;2(3):150-157.
