# Supplementary material for: The Effects of Perioperative Music Interventions in Pediatric Surgery: A Systematic Review and Meta-Analysis of Randomized Controlled Trials
Source: PLoS One. 2015 Aug 6;10(8):e0133608. doi: 10.1371/journal.pone.0133608 (PMC4527726; doi:10.1371/journal.pone.0133608)
Supplement: S5 File — Quality assessment of studies. (DOC) [file pone.0133608.s005.doc]

**S3 Risk of bias Quality assessment of studies: risk of bias as assessed by the data extractors**

|  | **Selection bias** | | **Performance bias** | **Detection bias** | **Attrition bias** | **Reporting bias** | **Overall risk of bias** |
| --- | --- | --- | --- | --- | --- | --- | --- |
|  | **Random sequence generation** | **Allocation concealment** | **Blinding participants and personnel** | **Blinding of outcome assessment** | **Incomplete outcome data** | **Selective reporting** |  |
| Bradt (2010) | Low | Unclear | Unclear | High | Unclear | Low | Moderate |
| Nilsson (2009) | Low | Low | Low | Low | Low | Low | Low |
| Hatem (2006) | High | Unclear | Low | Low | Unclear | Low | Moderate |
